# Alteration in Expression and Methylation of IGF2/H19 in Placenta and Umbilical Cord Blood Are Associated with Macrosomia Exposed to Intrauterine Hyperglycemia

**DOI:** 10.1371/journal.pone.0148399

**Published:** 2016-02-03

**Authors:** Rina Su, Chen Wang, Hui Feng, Li Lin, Xinyue Liu, Yumei Wei, Huixia Yang

**Affiliations:** 1 Department of Obstetrics and Gynecology, Peking University First Hospital, Beijing, China; 2 Pharmaceutical Outcomes and Policy, College of Pharmacy, University of Florida, Florida, United States of America; University of Rochester, UNITED STATES

## Abstract

**Objective:**

Macrosomia is one of the most common complications in gestational diabetes mellitus. Insulin-like growth factor 2 and H19 are two of the imprinted candidate genes that are involved in fetal growth and development. Change in methylation at differentially methylated region of the insulin-like growth factor 2 and H19 has been proved to be an early event related to the programming of metabolic profile, including macrosomia and small for gestational age in offspring. Here we hypothesize that alteration in methylation at differentially methylated region of the insulin-like growth factor 2 and H19 is associated with macrosomia induced by intrauterine hyperglycemia.

**Results:**

The expression of insulin-like growth factor 2 is significant higher in gestational diabetes mellitus group (GDM group) compared to normal glucose tolerance group (NGT group) both in umbilical cord blood and placenta, while the expression of H19 is significant lower in GDM group in umbilical cord blood. The expression of insulin-like growth factor 2 is significant higher in normal glucose tolerance with macrosomia group (NGT-M) compared to normal glucose tolerance with normal birthweight group (NGT-NBW group) both in placenta and umbilical cord blood. A model with interaction term of gene expression of IGF2 and H19 found that IGF2 and the joint action of IGF2 and H19 in placenta showed significantly relationship with GDM/NGT and GDM-NBW/NGT-NBW. A borderline significant association was seen among IGF2 and H19 in cord blood and GDM-M/NGT-M. The methylation level at different CpG sites of insulin-like growth factor 2 and H19 in umbilical cord blood was also significantly different among groups. Based on the multivariable linear regression analysis, the methylation of the insulin-like growth factor 2 / H19 is closely related to birth weight and intrauterine hyperglycemia.

**Conclusions:**

We confirmed the existence of alteration in DNA methylation in umbilical cord blood exposed to intrauterine hyperglycemia and reported a functional role in regulating gene associated with insulin-like growth factor 2/H19. Both of these might be the underlying pathogenesis of macrosomia. We also provided the evidence of strong associations between methylation of insulin-like growth factor 2/H19 and macrosomia induced by intrauterine hyperglycemia.

## Introduction

Gestational diabetes mellitus (GDM), which is defined as any degree of glucose intolerance with onset during pregnancy, affects 10~20% of all pregnancies [1, 2]. A growing body of research suggests that exposure to intrauterine hyperglycemia can increase the incidence of macrosomia, and these infants are prone to have obesity, abnormal glucose metabolism, hypertension and dyslipidemia in adulthood [3,4]. Many studies suggested that intrauterine hyperglycemia may increase maternal supply of carbohydrates leading to fetal hyperinsulinemia, and stimulation of the insulin sensitive tissue by hyperinsulinemia results in increased fetal growth [5,6], but the exact mechanism of macrosomia induced by intrauterine hyperglycemia environment of GDM is still not completely understood. In mammals, epigenetic reprogramming have been proposed to be involved in the development of human diseases caused by suboptimal environmental or nutritional factors [7]. Moreover, epigenetic abnormalities induced by GDM may be involved in metabolic diseases progression and fetal growth [8, 9]. We hypothesize that GDM increases the risk of macrosomia in offspring by altering epigenetic modification.

Imprinted genes play an important role in embryonic growth and development as well as in placental function [10]. Epigenetic disruption of imprinted genes due to early exposure to adverse environment was proposed to be associated with enhanced susceptibility to adult chronic diseases [11]. The maternally imprinted insulin-like growth factor II (IGF2) gene on chromosome 11p15.5 is one of the best-characterized epigenetically regulated loci. H19 is at 90 kb 3`of IGF2 and is reciprocally imprinted with respect to IGF2, regulating its imprinting and expression [12]. The methylation at differentially methylated region (DMR) of these imprinted genes is very sensitive to early developmental environment, but can be relatively stable throughout the course of individual’s life [13]. Some studies indicated that maternal factors, including GDM and obesity, regulate DNA methylation at the IGF2 and H19 DMRs [14–16], but the results are conflicting and inadequate. There is a lack of data proving the epigenetic change induced by prenatal environmental conditions in imprinted genes is among the mechanisms contributing to the association between GDM and macrosomia in the offspring.

In order to observe the associations between methylation of IGF2/H19 DMRs and macrosomia induced by intrauterine hyperglycemia, we analyzed the methylation levels of IGF2/H19 DMRs of umbilical cord blood of neonates born to normal and GDM.

## Materials and Methods

### Subjects

A total of 275 pregnant females attending the Department of obstetrics of Peking University First hospital were involved in this study. GDM was diagnosed through a diagnostic 2h 75g oral glucose tolerance test (OGTT) at 24–28th week of gestation by Chinese MOH 2011 criteria [17] when one of the following plasma glucose value was met or exceeded: 0 hour, 5.10mmol/L, 1 hour 10.0mmol/L and 2 hour 8.5mmol/L. Patients with any other complications were excluded (including but not limited to multiple gestation, pregnancy-induced hypertension, preeclampsia, infectious diseases, premature rupture of membrane, polyhydramnios, and fetal anomalies). Two hundred and seventy five infant-mother pairs were divided into GDM group(n = 136) and normal glucose tolerance (NGT) group (n = 139), then divided into four subgroups based on whether the birth weight of fetus > 4000gram: NGT with normal birth weight group (NGT-NBW group, n = 84), GDM with normal birth weight group (GDM-NBW group, n = 109), NGT with macrosomia group (NGT-M group, n = 55), and GDM with macrosomia group(GDM-M group, n = 27).

Clinical and demographic characteristics (including age, gestational age, lipid and pre-pregnancy weight and body mass index (BMI), antepartum weight and BMI, fetus weight, fetus height, and fetus head circumference) were collected through chart review. Oral informed consent was obtained from the participants and the data were analyzed anonymously. The study was approved by the clinical research ethics review board of Peking University First hospital (resolution 2014(823)).

### Tissues

Samples of placenta from patients were snipped respectively and tissue samples were snap frozen in liquid nitrogen and stored at -80°C before further processing. Umbilical cord blood samples were collected in Ethylene Diamine Tetraacetic Acid (EDTA)-treated tubes at delivery. Peripheral blood mononuclear cell (PBMCs) were isolated from 3ml anticoagulated cord blood using Lymphocytes Separation Medium (TBD science, Tianjin, China), the PBMCs were washed with phosphate-buffered saline at 4°C, the PBMCs was stored at -80°C until further test.

### Relative expression analysis in placenta and umbilical cord blood

Total RNA was isolated from 100 mg of placental tissues and PBMCs from all the patients using 1 mL of TRIzol Reagent (Invitrogen, USA) according to the manufacturer's protocol. Two microgram of total RNA was used as a template for reverse transcription. Oligo (dT) 10 primer and reverse transcriptase (Applied Biosystems, USA) were used according to the manufacturer's protocol. cDNA quantity was measured using Real-time PCR with the ABI PRISM 7500 sequence detector system and ABI PRISM 7500 TaqMan Master. PCR was performed in a final volume of 20 μl, consisting of diluted cDNA sample (2ug), 10x PCR buffer, 2.5uM dNTPs, 1xTaq enzyme, 1xROX, primers and primer probes optimized for each target gene and nuclease-free water. All samples were analyzed in triplicates. Primers were designed using Primer Express 3.0 software. The following primers were used, GAPDH: Sense:5’GAAGGTGAAGGTCGGAGTC3’,Antisense:5’GAAGATGGTGATGGGATTTC 3’, probe: 5’ CAAGCTTCCCGTTCTCAGCC 3’; IGF2: Sense: 5’ GTGCTACCCCCGCCAAGT 3’, Antisense: 5’ TGGACTGCTTCCAGGTGTCA 3’,probe:5’ACGTGTCGACCCCTCCGACCG3’;H19:Sense:5’GGCTCCCAGAACCCACAAC3’, Antisense:5’AGAGGGTTTTGTGTCCGGATT3’,probe:5’AAAGAAATGGTGCTACCCAGCTCAAGCC 3’.

### DNA methylation analysis in umbilical cord blood

30 samples from each group of NGT-NBW, NGT-M and GDM-NBW, 25 samples from GDM-M group were randomly chosen to detect the DNA methylation level. Total genomic DNA was isolated from 2ml anticoagulated cord blood using blood DNA midi kit (Omegar, USA), nucleic acid was examined qualitatively and quantitatively using electrophoresis and a spectrophotometer (NanoDrop 2000). The bisulphite treatment was followed by PCR amplification, fragmentation after reverse transcription and analysis on a mass spectrometer, according to the manufacture`s protocol (Sequenom, Inc, San Diego, USA). This generated mass signal patterns that were translated into quantitative DNA methylation levels of different CpG sites of the selected genes by MassARRAY EpiTYPER Analyzer software (Sequenom, Inc, San Diego, USA). Fragments containing one or more CpG sites were called CpG units. DNA methylation was assessed at two locations: in the promoter region encompassing -2238 to -1757 transcription start of H19 overlapping a CpG island, which is part of the H19 DMR, the methylation of 25 CpG sites was measured, and in the promoter region encompassing -2294 to -1731 transcription start of IGF2, which is part of the IGF2 DMR, methylation of 12 CpG sites was assessed. Data was processed and analyzed by the Sequenom MassARRAY Workstation software (version 3.3).

### Statistical analysis

One-way ANOVA and student t test was used to test differences in maternal or child characteristics between groups. Inter‑group differences were compared using the Post Hoc Tests. Linear mixed models were used to examine the associations between fetal birth weight and maternal biomarker concentrations and DNA methylation. This model was chosen as it accounts for correlation between CpG sites, incorporates relevant adjustments within the models and has the ability to accommodate missing data. Outliers per CpG (>3SD) were excluded from further analysis. All analyses were performed using SPSS software, version 20.0, values of *p* < 0.05 were considered to be statistically significant.

## Results

### General characteristics of subjects

Characteristics of the study subjects (n = 275 infant-mother pairs) are listed in Table 1. All the infants were full term. Mothers in GDM group had a significantly higher age, higher BMI during pre-pregnancy and antepartum, and higher lipid in first trimester compared to mothers in NGT group (*p*<0.05). Similar results were found in GDM-NBW group compared to NGT-NBW group. Fetus birth weight, height and head circumference showed no significant difference between GDM group and NGT group.

**Table 1:** Clinical Characteristics of the Study Subjects.

	GDM group			NGT group		
	GDM-NBW Group(n = 109)	GDM-M Group(n = 27)	Total n = 136	NGT-NBW Group(n = 84)	NGT-M Group(n = 55)	Total n = 139
Mother						
Age(yr)	32.19±3.42^**c**^	31.93±3.85^**d**^	32.14±3.50^**a**^	30.85±3.49	30.05±3.26	30.53±3.41
Gestation age(wk)	39.22±0.84	39.35±0.97	39.24±0.87	39.62±1.03	40.11±0.67	39.82±0.93
Prepregenancy BMI	23.62±6.73^**c**^	25.09±3.84	23.92±6.28	20.01±5.6	22.61±5.83^**c**^	21.05±5.81 ^**a**^
Antepartum BMI	29.41±4.25^**c**^	30.0±3.93	29.53±4.18 ^**a**^	26.89±2.55	29.31±3.35^**c**^	27.86±3.12
FPG(mmol/L)	5.47±0.09^**c**^	5.63±0.32 ^**d**^	5.50±0.09 ^**a**^	4.54±0.03	4.67±0.03	4.59±0.03
OGTT 1h (mmol/L)	9.89±0.18^**c**^	9.47±0.37 ^**d**^	9.82±0.37 ^**a**^	7.39±0.13	7.65±0.19	7.49±0.09
OGTT 2h(mmol/L)	8.31±0.17^**c**^	8.04±0.25 ^**d**^	8.27±1.61 ^**a**^	6.33±0.10	6.53±0.12	6.41±0.92
TG(mmol/L)	1.10±0.07	1.06±0.12	1.09±0.06 ^**a**^	0.90±0.09	0.83±0.06	0.87±0.06
TC(mmol/L)	4.07±0.08	4.22±0.17	4.10±0.08 ^**a**^	3.89±0.13	3.83±0.09	3.86±0.09
HDL(mmol/L)	1.79±0.29	1.49±0.08	1.72±0.22	1.58±0.05	1.59±0.04	1.59±0.04
LDL(mmol/L)	2.26±0.06	2.40±0.16 ^**d**^	2.29±0.07 ^**a**^	2.10±0.10	1.99±0.08	2.05±0.07
Fetus						
Fetal birth weight(g)	3408.4±326.60	4394.4±508.80 ^**b**^	3604.19±510.60	3346.5±336.50	4196.8±186.30^**c**^	3682.99±505.80
Fetal height(cm)	50.32±0.11	52.04±0.34	50.66±1.48	50.2±0.13	52.05±0.13	50.94±1.46
Fetal head circumference(cm)	33.86±0.08	34.91±0.19	34.07±0.99	33.86±0.11	34.69±0.10	34.18±1.01

Data are presented as Mean±SE. BMI; Body mass index, FPG; Fasting plasma glucose, OGTT; Oral glucose tolerance test, TG; Triglyceride, TC; Total cholesterol; HDL; High-density lipoprotein, LDL; Low density lipoprotein. Significance was determined by ANOVA.

^a^ p < 0.05 vs. NGT

^b^ p < 0.05 vs. GDM-NBW

^c^p < 0.05 vs. NGT-NBW

^d^P<0.05 vs. NGT-M

### The expression of IGF2/H19 in placenta and umbilical cord blood

**Fig 1:**
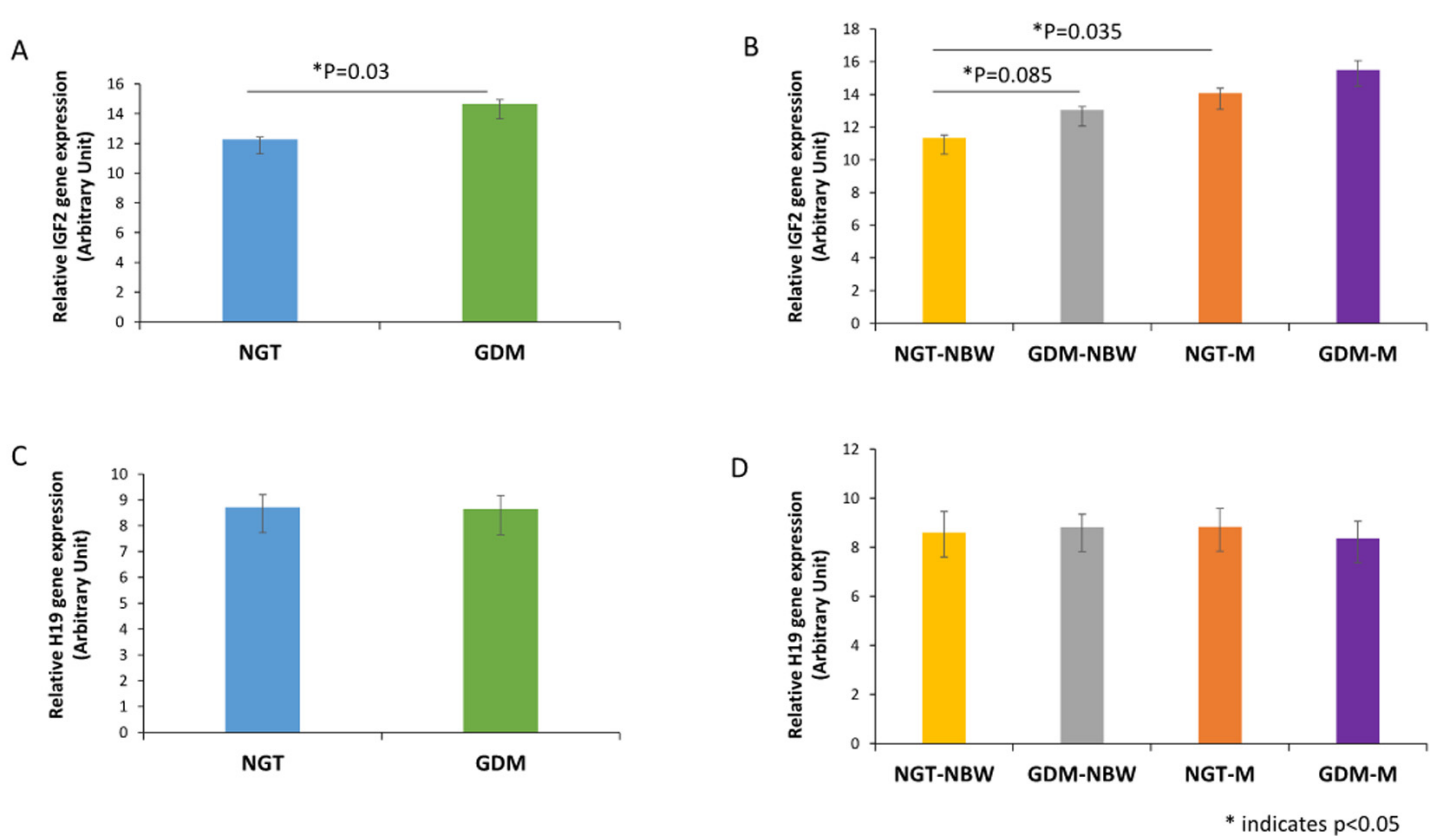
IGF2/H19 Expression in Placenta. (A)The expression of IGF2 in placenta was significantly higher in GDM group than that in NGT group. (B) The expression of IGF2 in placenta was significant higher in NGT-M group than NGT-NBW group. (C)The expression of H19 in placenta from GDM group had a trend to be decreased compared with control group. (D) There is no significant difference in placenta in each group. * indicates *p*<0.05.

**Fig 2:**
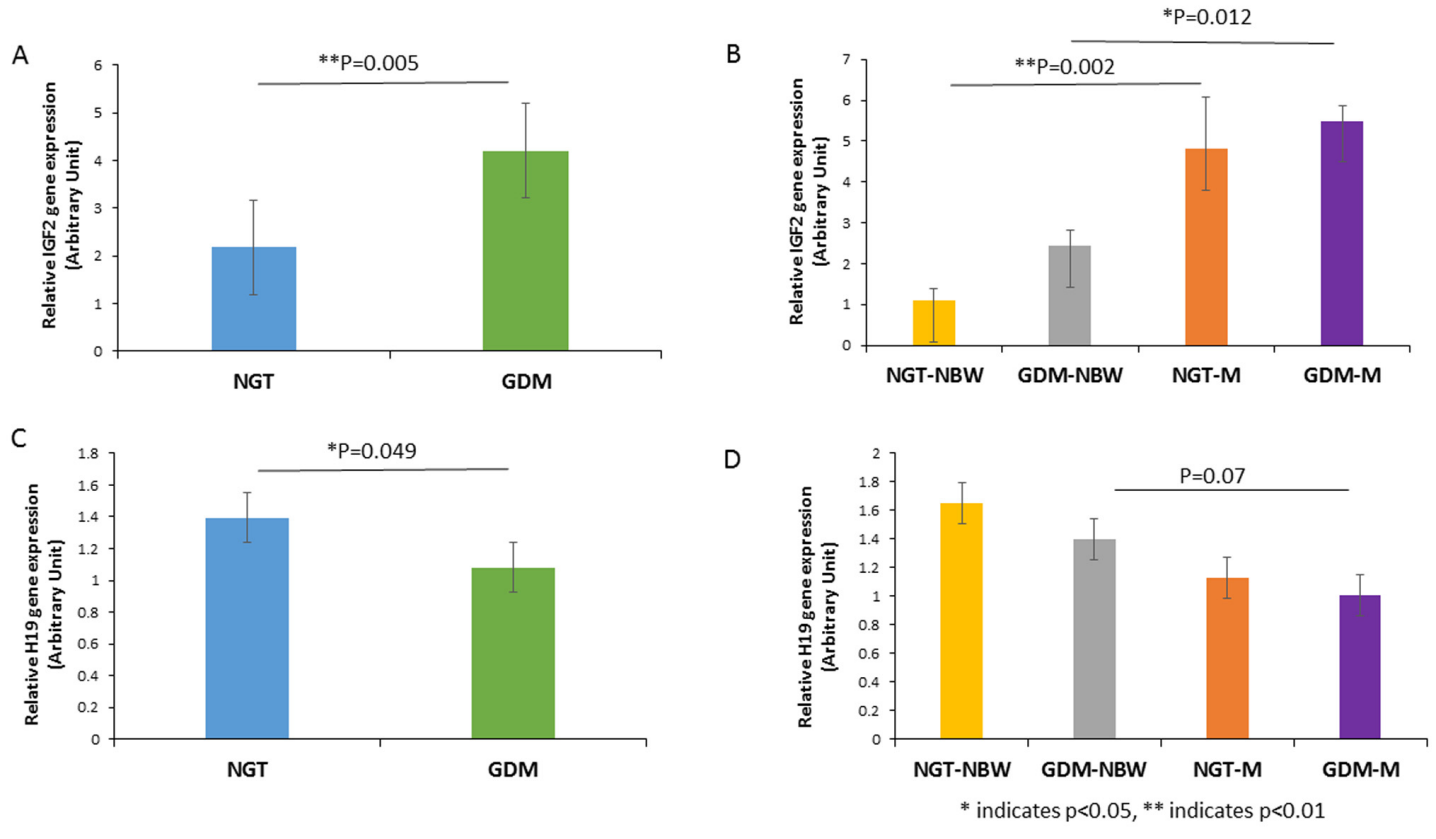
IGF2/H19 Expression in Umbilical Cord Blood. A, The expression of IGF2 in umbilical cord blood was significantly higher in GDM group than that in NGT group. (B) The expression of IGF2 in umbilical cord blood was significant higher in NGT-M group than that in NGT-NBW group. (C) The expression of H19 in umbilical cord blood was decreased significantly in GDM group compared to the NGT group. (D) The expression of H19 in umbilical cord blood had a trend decreased in NGT-M group and GDM-M group compared with normal group. * indicates *p*<0.05, ** indicates *p*<0.01.

The expression of IGF2 in placenta and umbilical cord blood was significantly higher in GDM group than that in NGT group (p = 0.030, p = 0.005 respectively, Fig 1A, Fig 2A). The expression of IGF2 in both placenta and umbilical cord blood was significant higher in NGT-M group than NGT-NBW group (*p* = 0.035, *p* = 0.002, respectively, Fig 1B, Fig 2B). The expression of IGF2 in placenta also had a trend to be higher in GDM-NBW group compared to the NGT-NBW group (*p* = 0.085, Fig 1B), but the expression of IGF2 in cord blood was significantly lower in GDM-M group compared to NGT-M group (*p* = 0.012, Fig 2B).

The expression of H19 in umbilical cord blood was significantly lower in GDM group than that in NGT group (p = 0.049, Fig 2C).The expression of H19 in cord blood was lower in GDM-M group compared with GDM-NBW group without statistical significance (P = 0.07, Fig 2D).

A model with interaction term of gene expression of IGF2 and H19 may be better for the data analysis namely Y = beta0+beta1(IGF2)+beta2(H19)+beta3(IGF2╳H19)(Table 2). We found that IGF2 and the joint action of IGF2 and H19 in placenta showed significantly relationship with GDM/NGT and GDM-NBW/NGT-NBW (*p* = 0.014, *p* = 0.011; *p* = 0.036, *p* = 0.044, respectively). A borderline significant association was seen among IGF2 and H19 in cord blood and GDM-M/NGT-M (*p* = 0.088, *p* = 0.080, respectively).

**Table 2:** The Coefficient Table of A Model with Interaction Term of IGF2 and H19 Expression.

	Beta 1(IGF2)		Beta 2(H19)		Beta 3(IGF2╳H19)	
	*Coeff*	*p value*	*Coeff*	*p value*	*Coeff*	*p value*
**Placenta**						
GDM/NGT	1.934	0.014*	1.181	0.070	0.947	0.036*
GDM-M/NGT-M	1.343	0.536	0.978	0.918	0.985	0.764
GDM-NBW/NGT-NBW	2.361	0.011*	1.216	0.073	0.937	0.044*
**Cord blood**						
GDM/NGT	0.855	0.096	0.816	0.342	1.054	0.345
GDM-M/NGT-M	0.510	0.088	4.596	0.080	0.740	0.166
**GDM-NBW/NGT-NBW**	**1.122**	**0.468**	**0.862**	**0.526**	**0.998**	**0.974**

Linear Mixed Model analysis

* indicates p<0.05

### The methylation levels of IGF2/H19 DMR in umbilical cord blood

**Fig 3:**
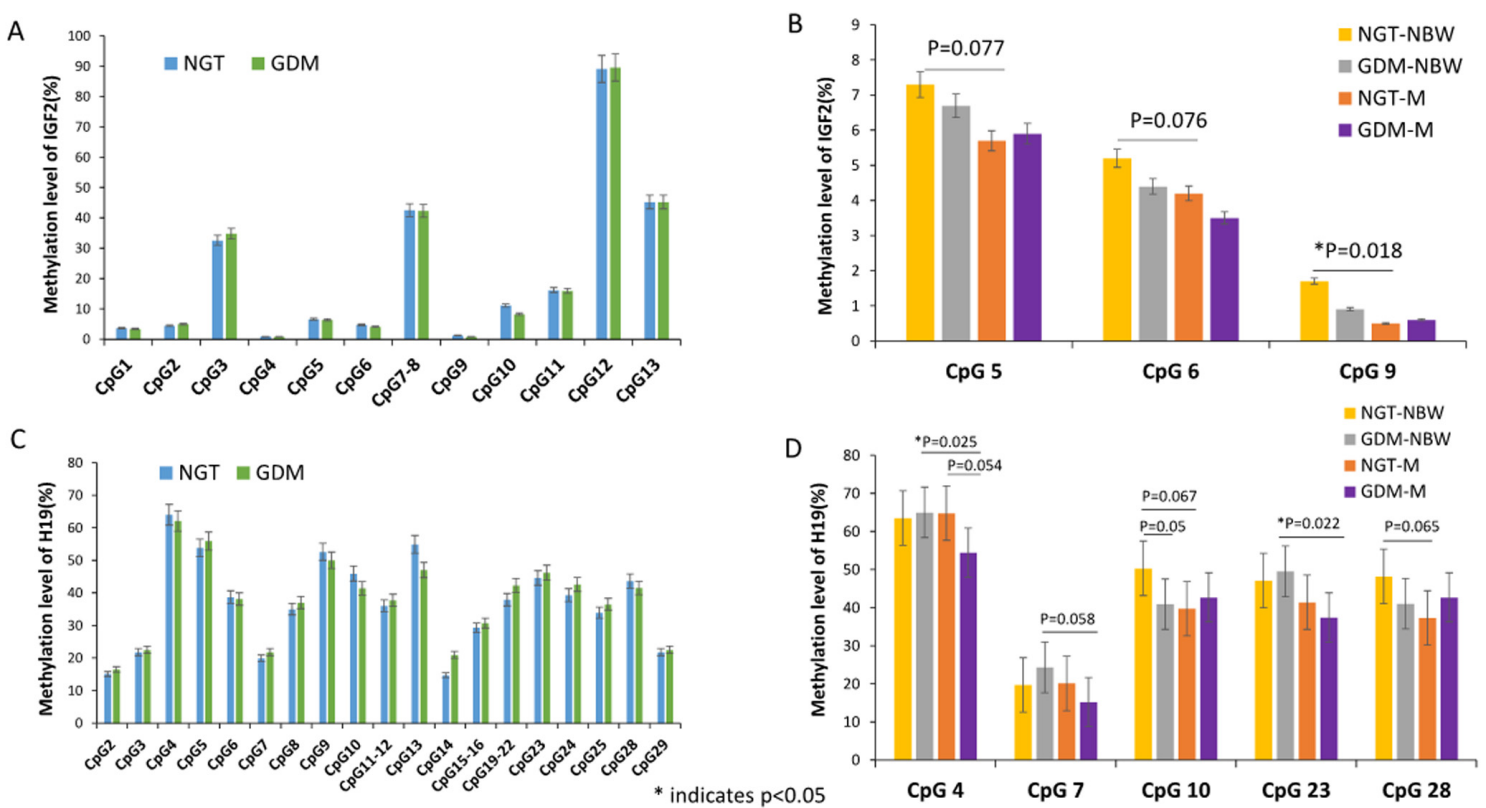
The Methylation Levels of IGF2/H19 Promoter Region in Umbilical Cord Blood. (A)The methylation level at sites 1,4,5,6,7~8,9,10 and 11 of IGF2 were lower in GDM group than NGT group. (B) The methylation level at CpG 5,6,and 9 of IGF2 were lower in NGT-M group than NGT-NBW group, CpG 9 was shown the significant difference between NGT-M group and NGT-NBW group and a borderline significant association for CpG 5 and 6 was found in NGT-M and NGT-NBW group. (C) The methylation level at sites 2,3,5,7,8,11~12,14,15~16,19~22,23,24,25 and 29 of H19 gene were higher in GDM group than NGT group. (D) The methylation level at CpG site 10 was shown a borderline significant difference between the GDM-NBW group and NGT-NBW group.The methylation level at CpG 4 and 23 were significantly lower in GDM-M group compared to the GDM-NBW group, and the CpG 7 was shown a borderline significant association between the GDM-M and GDM-NBW group. The methylation level at CpG site 4 was shown a borderline significant difference between the NGT-M group and the GDM-M group. A borderline significant decreased methylation level of CpG sites 10 and 28 of H19 DMR was found in NGT-M group compared to NGT-NBW group. * indicates p<0.05.

The methylation level of 12 CpG sites of IGF2 DMR and 25 CpG sites of H19 DMR quantified via Sequenom massarray from umbilical cord blood was shown in Table 3 and Fig 3.We found that the methylation level at CpG sites 1,4,5,6,7~8,9,10 and 11 of IGF2 DMR were lower in GDM group than NGT group. Furthermore, the methylation level at CpG sites 2,4,5,6,7~8,9,12 and 13 of IGF2 DMR were lower in GDM-M group than GDM-NBW group, but the methylation level did not show significant difference (*p*>0.05 for all).And the methylation level at CpG sites 4,5,6,9 and 12 of IGF2 DMR were lower in NGT-M group than that in NGT-NBW group, the methylation level of CpG site 9 of IGF2 DMR was significant lower in NGT-M group than that in NGT-NBW group(*p* = 0.018,Fig 3B) and a borderline significant decreased methylation level of CpG sites 5 and 6 of IGF2 DMR was found in NGT-M group compared to NGT-NBW group(*p* = 0.077,*p* = 0.076,respectively, Fig 3B).

**Table 3:** Average Methylation Level of IGF2/H19 Gene in Umbilical Cord Blood.

	GDM group			NGT group		
	GDM-NBW Group(n = 30)	GDM-M Group(n = 25)	Total n = 55	NGT-NBW Group(n = 30)	NGT-M Group(n = 30)	Total n = 60
	Methylation %(SD)	Methylation %(SD)	Methylation %(SD)	Methylation %(SD)	Methylation %(SD)	Methylation %(SD)
IGF2						
CpG 1	3.2(1.8)	3.9(2.4)	3.4(1.9)	3.2(1.5)	4.2(2.1)	3.6(1.8)
CpG 2	5.7(9.3)	2.9(4.3)	5.0(8.3)	4.4(6.4)	4.4(5.5)	4.4(6.0)
CpG 3	32.9(18.4)	39.9(11.0)	34.8(16.8)	30.3(14.4)	35.7(21.5)	32.6(17.8)
CpG 4	0.9(2.1)	0.9(2.6)	0.9(1.6)	0.9(1.7)	0.8(1.4)	0.8(2.2)
CpG 5	6.7(3.3)	5.9(4.3)	6.4(3.6)	7.3(2.9)	5.7(4.0) **^c^**	6.6(3.5)
CpG 6	4.4(2.2)	3.5(2.4)	4.1(2.2)	5.2(2.3)	4.2(1.7)	4.7(2.1)
CpG 7~8	42.5(2.3)	41.6(5.2)	42.3(3.4)	42.3(4.4)	42.7(2.5)	42.5(3.7)
CpG9	0.9(1.7)	0.6(1.2)	0.8(1.6)	1.7(2.2)	0.5(1.0) **^c^**	1.2(1.9)
CpG10	7.9(10.2)	9.1(11.3)	8.2(10.5)	9.9(12.8)	12.5(16.8)	11.1(14.5)
CpG11	15.7(4.1)	15.6(7.1)	15.9(5.0)	15.8(3.3)	15.6(3.4)	16.2(3.3)
CpG12	89.8(2.6)	89.1(2.8)	89.6(2.7)	89.3(2.7)	88.8(3.2)	89.1(2.9)
CpG13	45.4(1.3)	44.7(2.1)	45.8(1.6)	45.1(2.0)	45.2(1.5)	45.8(1.2)
H19						
CpG 2	18.8(17.6)	12.4(7.7)	16.5(15.7)	13.3(9.9)	17.5(12.7)	15.1(11.3)
CpG 3	23.1(9.2)	20.7(7.7)	22.5(0.08)	22.3(9.6)	21.0(10.1)	21.8(9.8)
CpG 4	65(15.1)	54.4(13.0) **^b^**	62.1(15.2)	63.5(16.0)	64.8(16.9)	64.0(16.3)
CpG 5	57.5(15.2)	51.4(12.7)	55.9(14.7)	55.9(12.1)	50.9(12.6)	53.8(12.5)
CpG 6	39.7(10.0)	33.9(11.6)	38.1(10.7)	40.6(13.8)	36.2(11.0)	38.7(12.7)
CpG 7	24.3(16.2)	15.2(10.4)	21.8(15.3)	19.7(12.0)	20.1(14.3)	19.9(12.9)
CpG 8	37.1(8.4)	36.9(11.0)	37.0(9.0)	35.5(11.3)	34.3(10.6)	35.0(10.9)
CpG 9	49.1(18.3)	52.7(18.6)	50.0(18.3)	52.2(13.3)	52.2(13.3)	52.6(14.6)
CpG 10	40.9(14.6) **^c^**	42.7(18.0)	41.4(15.4)	50.3(24.2)	39.7(16.3)	45.9(21.8)
CpG 11~12	37.5(8.6)	37.9(8.7)	37.7(8.5)	36.4(8.6)	35.4(9.8)	36.0(9.0)
CpG13	45.3(26.6)	52.1(20.1)	47.0(25.1)	56.2(30.9)	53.1(29.3)	54.9(30.0)
CpG14	22.3(23.4)	17.6 (24.4)	21.0(23.5)	15.3(16.4)	13.9(7.9)	14.7(13.4)
CpG15~16	31.2 (13.2)	29.2(11.3)	30.7(12.6)	29.5(12.7)	28.9(14.5)	29.3(13.4)
CpG19~22	44.6(24.4)	36.0(27)	42.3(25.2)	35.4(28.7)	41.2(24.5)	37.9(26.9)0)
CpG 23	49.5(17.4)	37.4(12.9) **^b^**	46.2(17.1)	47.1(15.3)	41.4(13.2)	44.6(14.6)
CpG 24	42.8(11.5)	42.1(8.8)	42.6(10.7)	40.9(10.3)	37.0(11.6)	39.3(10.9)
CpG 25	36.8(17.7)	35.9(11.6)	36.5(16.2)	36.7(13.3)	30.2(18.3)	33.9(15.8)
CpG28	41.0(14.6)	42.7(18.0)	41.5(15.4)	48.2(25.7)	37.3(17.2)	43.6(23.0)
CpG29	23.1(9.2)	20.7(7.7)	22.5(8.8)	22.3(9.6)	21.0(10.1)	21.8(9.8)

Significance was determined by ANOVA.

^b^ p < 0.05 vs. GDM-NBW

^c^p < 0.05 vs. NGT-NBW

Unlike the methylation level of IGF2, increased methylation level at CpG sites 2,3,5,7,8,11~12,14,15~16,19~22,23,24,25 and 29 of H19 DMR was found in GDM group compared to NGT group, and the else 12 sites of H19 DMR was shown lower methylation level in GDM group compared to the NGT group. The methylation level at CpG site 10 was shown a borderline significant difference between the GDM-NBW group and NGT-NBW group(*p* = 0.05).Moreover, the methylation level of CpG sites 2,3,4,5,6,7,8,14,15~16,19~22,23,24,25 and 29 of H19 gene were lower in GDM-M group than GDM-NBW group, the CpG 4 and 23 was shown significant difference between the two groups(*p* = 0.025, *p* = 0,022 respectively, Fig 3D), and the CpG site 7 was shown a borderline significant hypomethylation in GDM-M group compared to the GDM-NBW group (*p* = 0.058,Fig 3D).The methylation level at CpG site 4 was shown a borderline significant difference between the NGT-M group and the GDM-M group(*p* = 0.054,Fig 3D).The methylation level at CpG sites 2,4,7,9 and 19~22 of H19 DMR were higher in NGT-M group compared with NGT-NBW group, and the else CpG sites were lower in NGT-M group compared to NGT-NBW group. A borderline significant decreased methylation level of CpG sites 10 and 28 of H19 DMR was found in NGT-M group compared to NGT-NBW group (*p* = 0.067, *p* = 0,065 respectively, Fig 3D).

### Association among methylation level of IGF2/H19 DNA DMRs, birth weight and related factors

Multivariate linear regression was performed to examine the association among fetal birth weight, DNA methylation and other related factors (Table 4, Table 5). A negative association was seen between methylation of CpG sites 2, 10 and 12 of IGF2 DMR and fetal birth weight in offspring (*p* = 0.069, *p* = 0.049, *p* = 0.037, respectively), and a positive association was seen between CpG sites 15~16 of H19 DMR methylation and fetal birth weight (*p* = 0.032), yet methylation of CpG sites 23 of H19 DMR showed negative relationship with fetal birth weight (*p* = 0.017).

**Table 4:** Linear Regression of IGF2 Methylation on Fetal Birth Weight and Related Impact Indicators.

	CpG 1		CpG 2		CpG 6		CpG10		CpG 12		CpG 13	
Factor	*Coeff*	*p value*	*Coeff*	*p value*	*Coeff*	*p value*	*Coeff*	*p value*	*Coeff*	*p value*	*Coeff*	*p value*
Prepregenancy BMI	0.153	0.095	-0.118	0.056	-0.341	0.008**	-0.039	0.700	-0.089	0.392	-0.081	0.491
Antepartum BMI	0.101	0.341	-0.262	0.014*	-0.345	0.006**	0.053	0.594	-0.008	0.940	-0.001	0.991
FPG	0.092	0.396	-0.062	0.562	-0.302	0.020*	-0.030	0.767	0.073	0.48	-0.162	0.166
OGTT 1h	0.077	0.495	0.063	0.564	-0.160	0.244	-0.135	0.205	-0.082	0.442	0.278	0.026*
OGTT 2h	0.054	0.643	0.087	0.448	-0.024	0.866	-0.174	0.115	0.029	0.791	0.075	0.554
Fetal birth weight	0.056	0.654	-0.204	0.069	-0.174	0.218	-0.216	0.049*	-0.224	0.037*	-0.086	0.486

Linear Mixed Model analysis, FPG; Fasting plasma glucose, BMI; Body mass index, OGTT; Oral glucose tolerance test.

* indicates p<0.05

** indicates p<0.01

**Table 5:** Linear Regression of H19 Methylation on Fetal Birth Weight and Related Impact Indicators.

	CpG 4		CpG 14		CpG 15–16		CpG 19–22		CpG 23		CpG29	
Factor	*Coeff*	*p value*	*Coeff*	*p value*	*Coeff*	*p value*	*Coeff*	*p value*	*Coeff*	*p value*	*Coeff*	*p value*
Prepregenancy BMI	0.030	0.806	0.003	0.979	0.489	0.221	0.349	0.015*	-0.123	0.284	-0.436	0.228
Antepartum BMI	0.010	0.939	-0.006	0.958	0.634	0.113	0.393	0.006*	-0.134	0.242	-0.296	0.409
FPG	-0.314	0.021*	-0.539	0.000**	-0.359	0.373	0.275	0.057	-0.157	0.190	0.367	0.328
OGTT 1h	-0.277	0.050*	-0.276	0.015*	-0.501	0.237	-0.008	0.955	-0.007	0.954	0.163	0.677
OGTT 2h	-0.314	0.021*	-0.539	0.000**	-0.359	0.373	0.275	0.057	-0.157	0.190	0.367	0.328
Fetal birth weight	0.044	0.727	-0.089	0.407	0.886	0.032*	0.266	0.069	-0.285	0.017*	-0.629	0.090

Linear Mixed Model analysis, FPG; Fasting plasma glucose, BMI; Body mass index, OGTT; Oral glucose tolerance test.

* indicates p<0.05.

** indicates p<0.01.

A negative association was seen between DNA methylation at CpG sites 6 of IGF2 DMR and FPG of OGTT (*p* = 0.020), similar association was found between CpG site 13 of IGF2 DMR methylation and glucose of OGTT 1h. DNA methylation of CpG sites 4 and 14 of H19 DMR had a significant negative associated with FPG and glucose of OGTT 1h and 2h (*p*<0.05, respectively).

Analysis of the association among fetal birth weight related impact factor pre-pregnancy, anterpartum BMI and DNA methylation of IGF2/H19 DMRs was conducted. We found that DNA methylation of CpG sites 2 and 6 of IGF2 DMR showed negative relationship with pre-pregnancy and antepartum BMI (*p*<0.05).A positive association was seen among DNA methylation of CpG19~22 of H19 DMR, pre-pregnancy and antepartum BMI (*p*<0.05).

## Discussion

In this study, increased expression of IGF2 and decreased expression of H19 in placenta and cord blood were observed in fetuses of GDM group compared to NGT group. Moreover, expression of IGF2 was much higher in NGT-M group than that in NGT-NBW group. Increased expression of IGF2 may be related to high birth weight. A model with interaction term of gene expression of IGF2 and H19 found that IGF2 and the joint action of IGF2 and H19 in placenta showed significantly relationship with GDM/NGT and GDM-NBW/NGT-NBW, a borderline significant association was seen among IGF2 and H19 in cord blood and GDM-M/NGT-M. Our data suggest that altered expression of IGF2/H19 may be among the mechanisms linking maternal GDM and high risk of abnormal growth and development. The gene expression of IGF2 and the joint action of IGF2/H19 in placenta may be associated with maternal GDM. The gene expression of IGF2 and H19 in cord blood may be associated with macrosomia. There are some clinical and animal Zhang S, et al [18] found that elevated IGF2 concentrations were associated with higher birth weight after adjusting for maternal race/ethnicity, pre-pregnancy BMI, cigarette smoking, gestational diabetes, and infant sex in 300 pregnant women in USA, which suggests that IGF2 may continue to play an important role after birth. The results are consistent with our findings.

It is reported that DNA methylation associated changes in gene expression play a pivotal role in fetal and placenta development through two most widely-studied imprinted genes of H19 and IGF2, which are co-regulated within the same locus at human chromosome 11p15 [7,19,20]. IGF2 is highly expressed during prenatal development and its activity is regulated by genomic imprinting. The disrupted methylation of IGF2/H19 is associated with congenital growth disorders [21–23]. H19 DMR methylation may be involved in controlling IGF2 expression. In our study, the methylation level at CpG sites 5, 6 and 9 of IGF2 DMR was significantly lower in NGT-M group than that in NGT-NBW group. Furthermore, multivariate linear regression showed a negative association between CpG sites 2, 10 and 12 of IGF2 DMR methylation and fetal birth weight. Although there is no significant association between methylation level of H19 DMR and fetal high birth weight, a positive association was seen between CpG sites 15~16 of H19 DMR methylation and fetal birth weight. Our data indicate that increased methylation at H19 and decreased methylation at IGF2 might be associated with high birth weight. Consistent with our study, a study on the association of methylation in the IGF2/H19 region with body weight revealed that European and African-American children who are overweight or obese had higher levels of methylation in the H19DMR and lower level of methylation in IGF2 DMR at birth [23]. Previous studies also showed methylation level of H19 DMR was not associated with higher birth weight in infants [21, 22]. Nonetheless, not all studies demonstrate association of the IGF2/H19 methylation with birth weight. For example, Huang et al [24] found a negative association of IGF2/H19 methylation with head circumference but not birth weight or birth length. A study by Burris et al [8] also did not detect an association between birth weight and methylation in the imprinted IGF2/H19 region, but they suggest a potential gene–epigenetic interaction among a T-allele in the imprinting control region (ICR) of IGF2, methylation of IGF2 ICR and fetal growth.

Epigenetic mechanism has been proposed to involve in the link between environmental and nutritional factors and gene expression regulation. Many animal and human studies have illustrated that environment that restricts fetal growth might “program” future chronic disease through influencing epigenetic state in utero [25–26]. Most studies in this area focused on restricted intrauterine nutritional conditions and reported epigenetic modifications in intrauterine growth restriction (IUGR) model [27]. In our study we provide further evidence of intrauterine over-nutritional exposures.

Furthermore, we found relatively decreased methylation level of IGF2 DMR and increased methylation level of H19 DMR in GDM group compared to NGT group. Intrauterine hyperglycemia may affect expression of IGF2/H19 through altering methylation level of IGF2/H19. Consistent with our findings, Sireesha M et al [28] found that hyperglycemia upregulates insulin-like growth factors (IGFs), especially IGF2 in diabetes patients. Polymorphic variation in fetal IGF2 is associated with increased maternal glucose concentrations in pregnancy and this might be partially mediated by changes in placental IGF2 expression [29]. However, animal studies revealed hypermethylation at IGF2 in diabetic offspring. Ding et al [14] observed the expression of Igf2 and H19 was downregulated in pancreatic islets isolated from pups of GDM, and it may be caused by hypermethylation status of the differentially methylated region. The differences in maternal disorder (GDM versus diabetes) and birth weight are the possible factors for the different methylation in IGF2 in human and mice.

Of interest, the methylation level of H19 DMR were lower in GDM-M group than GDM-NBW group, especially the CpG sites 4, 7 and 23. Multivariate linear regression showed that methylation of CpG sites 23 of H19 DMR had significantly negative relationship with fetal birth weight. Moreover, DNA methylation of CpG sites 4 and 14 of H19 DMR had a significant negative association with FPG and glucose of OGTT.1h and 2h. Shao et al [30] reported that expression of IGF2 in fetuses from diabetic mice was 0.65-fold of the control counterparts and the methylation level of the H19-IGF2 imprint control region was 19.1% higher in diabetic dams and body weight of pups born to diabetic dams was 26.5% lower than control dams. It is suggested that hypermethylation of imprint control region (same as DMR) is related to low birth weight of diabetic mothers. In our study we found that hypomethylation of some CpG sites in imprint control region is related to high birth weight of GDM. The mechanisms of the decreased methylation level of H19 are unknown, but differential histone modification has been reported at many imprinted domains.

To our knowledge, there are few studies analyzing the relationship between high birth weight induced by hyperglycemia and alteration of DNA methylation level of imprinting gene. Our results indicate that GDM can affect fetal development by means of altered expression of imprinted genes. The modified genomic DNA methylation status of imprinting genes may account for the change in gene expression.

How intrauterine environmental and nutrition factors affect birth weight and outcomes remains largely unknown and epigenetic data may fill some of these gaps. Elucidating the epigenetic mechanism in pathophysiology of fetal growth will improve perinatal outcomes and be helpful for the obstetrician control birth defects.

The development of embryos exposed to intrauterine hyperglycemia is complex [31]. Hyperglycemia can alter imprinting gene expression, resulting in aberrant cell signaling. The placenta is a fetal-maternal endocrine organ responsible for maintaining and regulating pregnancy stages. Throughout the in utero development of the fetus, the placenta is crucial to growth of the fetus. The placenta can be considered an important and rather accessible record of in utero exposures and pathology. In future experiments, the study of the methylation level in placenta which is directly responsible for fetal growth might be useful to clarify the relationship between DNA methylation and birth weight [32, 33]. The relationship between altered methylation level of specific sites of IGF2 and H19 and hyperglycemia should be investigated in depth in animal and cell studies.

## Conclusion

GDM can affect fetal development by increasing fetal birth weight. We confirmed the existence of alteration in DNA methylation in umbilical cord blood exposed to intrauterine hyperglycemia and reported a functional role in regulating gene associated with IGF2/H19. Both of these might be the underlying pathogenesis of macrosomia. We also provided the evidence of strong associations between methylation of IGF2/H19 DMRs and macrosomia induced by intrauterine hyperglycemia.

## Abbreviations

GDM: gestational diabetes mellitus
IGF2: insulin-like growth factor 2
DMR: differentially methylated region
OGTT: oral glucose tolerance test
PBMCs: Peripheral blood mononuclear cell
FPG: fasting plasma glucose
BMI: body mass index

## References

[1] Zhu WW, Yang HX, Wei YM, Yan J, Wang ZL, Li XL, et al Evaluation of the value of fasting plasma glucose in the first prenatal visit to diagnose gestational diabetes mellitus in china.Diabetes Care. 2013; 6:586–90. 10.2337/dc12-1157 PMC3579369 23193214

[2] Mpondo BC, Ernest A, Dee HE.Gestational diabetes mellitus: challenges in diagnosis and management.J Diabetes Metab Disord. 2015;12;14:42. 10.1186/s40200-015-0169-7 PMC4430906 25977899

[3] Franks PW, Looker HC, Kobes S, Touger L, Tataranni PA, Hanson RL, et al Gestational glucose tolerance and risk of type 2 diabetes in young Pima Indian offspring. Diabetes, 2006;55: 460–465. 16443781 10.2337/diabetes.55.02.06.db05-0823

[4] Manderson JG, Mullan B, Patterson CC, Hadden DR, Traub AI, McCance DR.Cardiovascular and metabolic abnormalities in the offspring of diabetic pregnancy. Diabetologia,2002;45: 991–996. 12136397 10.1007/s00125-002-0865-y

[5] Parretti E, Mecacci F, Papini M, Cioni R, Carignani L, Mignosa M, La Torre P, et al Third-trimester maternal glucose levels from diurnal profiles in nondiabetic pregnancies: correlation with sonographic parameters of fetal growth.Diabetes Care, 2001; 24:1319–23. 11473063 10.2337/diacare.24.8.1319

[6] Suliman SG, Stanik J, McCulloch LJ, Wilson N, Edghill EL, Misovicova N, et al Severe insulin resistance and intrauterine growth deficiency associated with haploinsufficiency for INSR and CHN2: new insights into synergistic pathways involved in growth and metabolism.Diabetes. 2009;8:2954–2961. 10.2337/db09-0787 PMC2780873 19720790

[7] Chatterjee A, Eccles MR. DNA methylation and epigenomics: new technologies and emerging concepts. Genome Biol. 2015; 16:103 10.1186/s13059-015-0674-5 25990550 PMC4438343

[8] Burris HH, Braun JM, Byun HM, Tarantini L, Mercado A, Wright RJ, et al Association between birth weight and DNA methylation of IGF2, glucocorticoid receptor and repetitive elements LINE-1 and Alu. Epigenomics. 2013; 5:271–81. 10.2217/epi.13.24 23750643 PMC3787720

[9] Allard C, Desgagné V, Patenaude J, Lacroix M, Guillemette L, Battista MC, et al Mendelian randomization supports causality between maternal hyperglycemia and epigenetic regulation of leptin gene in newborns.Epigenetics. 2015;10:342–351. 10.1080/15592294.2015.1029700 25800063 PMC4622547

[10] Constância M, Hemberger M, Hughes J, Dean W, Ferguson-Smith A, Fundele R, Stewart F, Kelsey G, Fowden A, Sibley C, Reik W. Placental-specific IGF-II is a major modulator of placental and fetal growth. Nature. 2002;417:945–948. 12087403 10.1038/nature00819

[11] Waterland RA, Jirtle RL: Early nutrition, epigenetic changes at transposons and imprinted genes, and enhanced susceptibility to adult chronic diseases. Nutrition 2004;20:63–68. 14698016 10.1016/j.nut.2003.09.011

[12] Bell AC, Felsenfeld G. Methylation of a CTCF-dependent boundary controls imprinted expres sion of the Igf2 gene. Nature 2000;405:482–485. 10839546 10.1038/35013100

[13] Tobi EW, Heijmans BT, Kremer D, Putter H, de Waal HA D-v, Finken MJ, Wit JM, Slagboom PE: DNA methylation of IGF2, GNASAS, INSIGF and LEP and being born small for gestational age. Epigenetics 2011;6:171–176. 20930547 10.4161/epi.6.2.13516 PMC3278785

[14] Ding GL, Wang FF, Shu J, Tian S, Jiang Y, Zhang D, et al Transgenerational glucose intolerance with Igf2/H19 epigenetic alterations in mouse islet induced by intrauterine hyperglycemia. Diabetes. 2012;61:1133–1142. 10.2337/db11-1314 22447856 PMC3331740

[15] Xie T, Chen M, Gavrilova O, Lai EW, Liu J, Weinstein LS.Severe obesity and insulin resistance due to deletion of the maternal Gsalpha allele is reversed by paternal deletion of the Gsalpha imprint control region.Endocrinology. 2008;149:2443–2450. 10.1210/en.2007-1458 18202131 PMC2329281

[16] Claycombe KJ, Uthus EO, Roemmich JN, Johnson LK, Johnson WT. Prenatal low-protein and postnatal high-fat diets induce rapid adipose tissue growth by inducing Igf2 expression in Sprague Dawley rat offspring. J Nutr. 2013; 143:1533–1539. 10.3945/jn.113.178038 23946348

[17] Yang HX.Diagnostic criteria for gestational diabetes mellitus (WS 331–2011).Chin Med J (Engl). 2012;125:1212–1213. 22613589

[18] Zhang S, Zhai G, Wang J, Shi W, Zhang R, Chen C. IGF-II expression and methylation in small for gestational age infants. J Pediatr Endocrinol Metab. 2015; 28:613–618. 10.1515/jpem-2014-0269 25503665

[19] LaRocca J, Binder AM, McElrath TF, Michels KB. The impact of first trimester phthalate and phenol exposure on IGF2/H19 genomic imprinting and birth outcomes. Environ Res. 2014;133:396–406. 10.1016/j.envres.2014.04.032 24972507 PMC4155603

[20] Algar EM, St Heaps L, Darmanian A, Dagar V, Prawitt D, Peters GB, et al Paternally inherited submicroscopic duplication at 11p15.5 implicates insulin-like growth factor II in overgrowth and Wilms' tumorigenesis. Cancer Res. 2007;67:2360–2365. 17325026 10.1158/0008-5472.CAN-06-3383

[21] Court F, Martin-Trujillo A, Romanelli V, Garin I, Iglesias-Platas I, Salafsky I, et al Genome-wide allelic methylation analysis reveals disease-specific susceptibility to multiple methylation defects in imprinting syndromes.Hum Mutat. 2013;34:595–602. 10.1002/humu.22276 23335487

[22] St-Pierre J, Hivert MF, Perron P, Poirier P, Guay SP, Brisson D, et al IGF2 DNA methylation is a modulator of newborn’s fetal growth and development. Epigenetics. 2012;7:1125–1132. 10.4161/epi.21855 22907587 PMC3469454

[23] Hoyo C, Fortner K, Murtha AP, Schildkraut JM, Soubry A, Demark-Wahnefried W, et al Association of cord blood methylation fractions at imprinted insulin-like growth factor 2 (IGF2), plasma IGF2, and birth weight. Cancer Causes Control. 2012; 23:635–645. 10.1007/s10552-012-9932-y 22392079 PMC3313040

[24] Perkins E, Murphy SK, Murtha AP, Schildkraut J, Jirtle RL, Demark-Wahnefried W, et al Insulin-like growth factor 2/H19 methylation at birth and risk of overweight and obesity in children. J Pediatr. 2012;161:31–39. 10.1016/j.jpeds.2012.01.015 22341586 PMC3360130

[25] Huang RC, Galati JC, Burrows S, Beilin LJ, Li X, Pennell CE, et al DNA methylation of the IGF2/H19 imprinting control region and adiposity distribution in young adults. Clin Epigenetics.2012; 4:21 10.1186/1868-7083-4-21 23148549 PMC3507742

[26] Chen Danqing, Zhang Aiping, Fang Min, Fang Rong, Ge Jiamei, Jiang Yuan, et al Increased methylation at differentially methylated region of GNAS in infants born to gestational diabetes. BMC Medical Genetics 2014;15:108 10.1186/s12881-014-0108-3 25269528 PMC4411875

[27] Koukoura O, Sifakis S, Soufla G, Zaravinos A, Apostolidou S, Jones A, et al Loss of imprinting and aberrant methylation of IGF2 in placentas from pregnancies complicated with fetal growth restriction. Int J Mol Med. 2011;28:481–7 10.3892/ijmm.2011.754 21805044

[28] Sireesha M, Sambasivan V, Kumar VK, Radha S, Raj AY, Qurratulain H. Relevance of insulin-like growth factor 2 in the etiopathophysiology of diabetic nephropathy: possible roles of phosphatase and tensin homolog on chromosome 10 and secreted protein acidic and rich in cysteine as regulators of repair. J Diabetes. 2009;1:118–24. 10.1111/j.1753-0407.2009.00025.x 20929508

[29] Petry CJ, Seear RV, Wingate DL, Manico L, Acerini CL, Ong KK, et al Associations between paternally transmitted fetal IGF2 variants and maternal circulating glucose concentrations in pregnancy. Diabetes. 2011;60:3090–3096. 10.2337/db11-0689 21926269 PMC3198064

[30] Shao WJ, Tao LY, Gao C, Xie JY, Zhao RQ. Alterations in methylation andexpression levels of imprinted genes H19 and Igf2 in the fetuses ofdiabetic mice. Comp Med 2008; 58:341–346. 18724775 PMC2706039

[31] Ornoy A, Reece EA, Pavlinkova G, Kappen C, Miller RK.Effect of maternal diabetes on the embryo, fetus, and children: congenital anomalies, genetic and epigenetic changes and developmental outcomes.Birth Defects Res C Embryo Today. 2015;105:53–72. 10.1002/bdrc.21090 25783684

[32] Ideraabdullah FY, Thorvaldsen JL, Myers JA, Bartolomei MS. Tissue-specific insulator function at H19/Igf2 revealed by deletions at the imprinting control region. Hum Mol Genet. 2014; 23:6246–6259. 10.1093/hmg/ddu344 24990148 PMC4222363

[33] Finer S, Mathews C, Lowe R, Smart M, Hillman S, Foo L, et al Maternal gestational diabetes is associated with genome-wide DNA methylation variation in placenta and cord blood of exposed offspring.Hum Mol Genet. 2015;24:3021–3029. 10.1093/hmg/ddv013 25634562

